# Prone lateral transpsoas interbody fusion with robotic assistance

**DOI:** 10.3171/2025.4.FOCVID2515

**Published:** 2025-07-01

**Authors:** Joseph M. Abbatematteo, Katriel E. Lee, Juan P. Giraldo, Laura A. Snyder

**Affiliations:** Department of Neurosurgery, St. Joseph’s Hospital and Medical Center, Barrow Neurological Institute, Phoenix, Arizona

**Keywords:** lateral access approach, lateral transpsoas approach, minimally invasive spine surgery, percutaneous pedicle screws

## Abstract

This video demonstrates robotic assistance for performing single-position prone lateral transpsoas interbody fusion with posterior percutaneous screws. The ability to preoperatively plan the screws and interbody devices may help surgeons improve accuracy as well as increase the ease of performing these single-position minimally invasive procedures.

The video can be found here: https://stream.cadmore.media/r10.3171/2025.4.FOCVID2515

## Transcript

Prone lateral transpsoas interbody fusion with robotic assistance. This video will demonstrate the use of robotic assistance for performing minimally invasive single-position prone lateral transpsoas interbody fusion with posterior percutaneous screw fixation.

### 0:40

A patient in his early 60s presented with back pain when standing and bending, as well as symptoms of neurogenic claudication. On physical examination, the patient demonstrated 5/5 strength in all bilateral lower extremity muscle groups. The patient could not tolerate medication such as gabapentin or pregabalin. Epidural steroid injections and physical therapy had helped minimally for his pain.

### 1:10

Preoperative MRI showed degenerative disc disease at L3–4 and L4–5, with moderate canal stenosis at L3–4, severe stenosis at L4–5, and grade I spondylolisthesis at L4–5.

### 1:26

Preoperative flexion-extension radiographs showed mobile grade I anterolisthesis of L4 on L5.

### 1:37

Preoperative standing scoliosis radiographs showed lumbar dextroscoliosis, but coronal and sagittal vertical alignment was within normal limits.

### 1:47

After review of the patient’s history, physical examination findings, and imaging, as well as discussion with the patient, I decided to proceed with a right prone lateral transpsoas interbody fusion at L3–4, L4–5 with use of morselized allograft and posterior percutaneous screw fixation with robotic assistance. This would indirectly decompress the patient’s nerve roots as well as reduce and fixate his mobile L4–5 spondylolisthesis.

### 2:20

I prefer to use the prone lateral position instead of the single-position lateral position to facilitate segmental lordosis and allow ease of access for posterior decompression and posterior screw placement.^1^

### 2:35

Preoperative computed tomography (CT) was performed 2 days before the surgery, and the CT scan data was delivered to the robotic device (ExcelsiusGPS, Globus Medical). Screw and interbody trajectories and sizes were planned prior to surgery. On the day of surgery, the patient was positioned on a Jackson table with a bed positioner with pads pushing against the chest and the right iliac crest to angulate open the space between the right ribs above the pelvic crest for the right prone lateral incision.

### 3:09

These chest and pelvic bolsters create coronal bending and help move the iliac crest away from the working corridor when accessing the L4–5 disc space.

### 3:20

The patient’s back and right lateral side were prepped and draped in a usual fashion, and 2 small incisions were made over the bilateral posterosuperior iliac spine (PSIS). The dynamic reference base array (DRB) was placed into one PSIS, and the surveillance marker was placed into the other PSIS. The CT scan was coregistered with the patient’s anatomy using anteroposterior and lateral radiographs with a C-arm.^2^ The robot arm travels to the planned screw trajectories, and an incision can be made that splits the difference between them and allows all 3 screws and their percutaneous towers to pass. Sequential screws are then placed with the same workflow: first burr, then drill, then screw, working toward the reference array, all with the navigational guidance of the robotic system through the robot arm on the same trajectory.^3^

### 4:18

Anteroposterior and lateral fluoroscopy is performed after placement of the screws to verify that they are in good position. After all 6 screws are placed, stabilizer cuffs are placed over the percutaneous screw towers to align the screw heads to pass a rod. The rod will be placed after the interbody devices are placed, and the patient is placed back into a neutral position in the coronal plane later in the case.

### 4:47

Attention is then turned to the right lateral abdomen above the iliac crest. The navigated dilator can be used to plan an incision to enter the retroperitoneal space in the trajectory of the planned interbody disc space. Once through the retroperitoneal space, the first dilator can be used to dilate through the lateral transpsoas muscle of the planned interbody disc space. A clip for careful neurophysiologic or electromyographic monitoring of the dilator is attached to the back of the dilator to ensure that there are no plexus nerves too close nearby.^4^ A K-wire is placed to hold the ideal location of the dilator in the disc space.

### 5:31

Sequential dilators are placed again with clip electromyography stimulation to prevent nerve injury.

### 5:38

The retractor is placed over the dilators and locked into position over the desired disc space with the robotic arm attachment.

### 5:47

A shim can be placed to further hold the retractor at the correct level in the anteroposterior direction. The disc annulus is cut using a retractable blade to avoid injury when coming in and out of the retractor. Care is taken not to cut too close to the posterior shim because this may dislodge the retractor.^5^

### 6:07

The navigated Cobb elevators can be used to disrupt the contralateral disc osteophyte at the level of the disc space. The disc can then be removed with a combination of pituitary rongeurs, Kerrison rongeurs, and curettes. Many of these instruments can be navigated with visualization on the robotic device.^6^

### 6:29

Trials can be navigated into the desired disc space to verify that the goal interbody size will fit appropriately.

### 6:36

A titanium interbody graft packed with morselized allograft is placed under an anteroposterior and lateral fluoroscopic guidance as well as navigational robotic guidance.

### 6:49

The interbody can be expanded if desired to further fill the disc space.^7^

### 6:54

The starting height of the interbody is 7 mm, and it expands to 14 mm. The lordosis achieved by the interbody is 3° to 15° based on the level of expansion.

### 7:07

Anteroposterior and lateral fluoroscopy is performed after placement of each interbody to verify that each is in good position, and then the retractor can be removed.

### 7:20

Dilation was performed again in the retroperitoneal space over the next disc space in a new location on the psoas muscle. The navigated disc preparation and interbody placement was then repeated at the next level.

### 7:34

I have found that the loss of accuracy is minimal when proceeding between two interbodies. However, if loss of accuracy is significant, reregistration can be performed with anteroposterior and lateral fluoroscopy.

### 7:50

The lateral retractor can then be removed from the lateral incision once all interbody devices are placed. Hemostasis and irrigation of this incision can occur while the rods are being tunneled through the posterior percutaneous screw towers on either side. The patient is placed back into a neutral position in the coronal plane before the rods are locked into place with set screws.

### 8:16

Postoperative scoliosis radiographs show the interval placement of the L3–5 interbody devices as well as the patient’s posterior percutaneous screws.

### 8:26

The patient’s bilateral leg pain had improved by his 2-week postoperative visit, and his preoperative back pain had improved by his 6-week postoperative visit.

## Acknowledgments

We thank the staff of Neuroscience Publications at Barrow Neurological Institute for assistance with manuscript and video preparation.

## Disclosures

Dr. Snyder reported consulting fees from Globus outside the submitted work.

## Author Contributions

Primary surgeon: Snyder. Assistant surgeon: Abbatematteo. Editing and drafting the video and abstract: Snyder, Abbatematteo, Lee. Critically revising the work: all authors. Reviewed submitted version of the work: Snyder, Lee. Approved the final version of the work on behalf of all authors: Snyder. Supervision: Snyder.

## Supplemental Information

### Patient Informed Consent

The necessary patient informed consent was obtained in this study.
